# Action-based effects on music perception

**DOI:** 10.3389/fpsyg.2013.01008

**Published:** 2014-01-03

**Authors:** Pieter-Jan Maes, Marc Leman, Caroline Palmer, Marcelo M. Wanderley

**Affiliations:** ^1^Department of Music Research, McGill UniversityMontreal, QC, Canada; ^2^Department of Musicology, Ghent UniversityGhent, Belgium; ^3^Department of Psychology, McGill UniversityMontreal, QC, Canada

**Keywords:** embodied music cognition, common coding theory, sensory-motor association learning, dynamical systems, internal model

## Abstract

The classical, disembodied approach to music cognition conceptualizes action and perception as separate, peripheral processes. In contrast, embodied accounts of music cognition emphasize the central role of the close coupling of action and perception. It is a commonly established fact that perception spurs action tendencies. We present a theoretical framework that captures the ways in which the human motor system and its actions can reciprocally influence the perception of music. The cornerstone of this framework is the common coding theory, postulating a representational overlap in the brain between the planning, the execution, and the perception of movement. The integration of action and perception in so-called internal models is explained as a result of associative learning processes. Characteristic of internal models is that they allow intended or perceived sensory states to be transferred into corresponding motor commands (inverse modeling), and vice versa, to predict the sensory outcomes of planned actions (forward modeling). Embodied accounts typically refer to inverse modeling to explain action effects on music perception (Leman, 2007). We extend this account by pinpointing forward modeling as an alternative mechanism by which action can modulate perception. We provide an extensive overview of recent empirical evidence in support of this idea. Additionally, we demonstrate that motor dysfunctions can cause perceptual disabilities, supporting the main idea of the paper that the human motor system plays a functional role in auditory perception. The finding that music perception is shaped by the human motor system and its actions suggests that the musical mind is highly embodied. However, we advocate for a more radical approach to embodied (music) cognition in the sense that it needs to be considered as a dynamical process, in which aspects of action, perception, introspection, and social interaction are of crucial importance.

## 1. Introduction

Music is known to be a powerful medium that evokes body movements in listeners, ranging from tapping the feet, shaking the head, swaying the arms and hips, to more sophisticated forms of free or stylized dance. Research has shown that these body movements often reflect the performer's movements from which the music originated (Leman et al., 2009; Godøy and Leman, 2010), certain aspects of the melody, harmony, rhythm and timbre (Maes et al., 2010; Naveda and Leman, 2010; Toiviainen et al., 2010; Burger et al., 2013; Leman et al., 2013), or even the listeners' mood (Van Dyck et al., 2013). These and similar studies importantly indicate that the listeners' musical mind (attention, intention, mood, feelings, etc.) can be accessed through body movement, without the need for symbolic representations like language or musical scores. However, despite the explicit focus on the human body and body movements, these and similar studies do not consider the musical mind as being fundamentally embodied. The findings do not exclude the possibility that movement responses to music are mere peripheral epiphenomena resulting from central cognitive processes. Only recently have studies started to emerge, demonstrating how the musical mind can be shaped by the human motor system and the movements it produces (Phillips-Silver and Trainor, 2005, 2007; Repp and Knoblich, 2009; Sedlmeier et al., 2011; Iordanescu et al., 2013; Loehr, 2013; Manning and Schutz, 2013; Maes and Leman, 2013; Timm et al., 2013). This line of research reflects an important paradigm shift within cognitive science. The classical view, inspired by the developments of computer science and artificial intelligence in the 1950s–1960s, pertains to an “information processing” approach that considers a strictly unidirectional information flow from perception (input) to cognition (central processing unit) to action (output) (Neisser, 1967; Laske, 1974; Fodor, 1975; Pylyshyn and Demopoulos, 1986; Massaro, 1990). Accordingly, sensory information received from the external world is perceived, translated into a syntactic code of meaningful symbols, and processed according to a systematic set of rules. Then, body movements and other sorts of behavior are considered as mere outcomes of these higher-level, formal symbol manipulations. Hence, in this classical view of cognition, perception and action are completely separated from each other, and are outside central cognition [what Hurley (2001) describes as the “sandwich model of cognition”]. This classical model is obsolete, as research shows that perception and action are strongly intertwined and can mutually exert influence on each other. In what became the *embodied* cognition theory, the human body - with its perceptual and motor systems - and its interaction with the outside world, became central to human cognition (Varela et al., 1991; Leman, 2007; Chemero, 2009; Krueger, 2009; Glenberg, 2010; Shapiro, 2010). Within this framework of embodied cognition, the common coding theory (Prinz, 1990, 1997; Hommel et al., 2001) has been an influential theory postulating a close coupling between perception and action. Although the theory is not readily falsifiable, it provides a general framework for developing more detailed and testable explanatory models (cf. Hommel et al., 2001). In essence, the theory states that the planning or execution of an action, and the mere perception of the (multi-)sensory consequences of that action, are similarly represented (coded) in the brain, thereby recruiting both sensory and motor brain areas. Important in this theory is that the integration of motor and sensory representations leads to internal models of the relationship between the body and the external environment, which can contain inverse and forward components (Wolpert et al., 1995). Inverse models represent an information flow from perception to action, in the sense that they allow the system to estimate from incoming sensory information the corresponding motor commands required to generate that specific sensory state [cf. Rizzolatti et al. (2001): direct-matching hypothesis]. In contrast, forward models represent an information flow from action to perception, in the sense that they allow to predict the likely sensory outcome of a planned or executed action (Davidson and Wolpert, 2005; Bubic et al., 2010; Waszak et al., 2012). Currently, the idea is gaining consensus that the combination of inverse and forward modeling processes guides people's interaction with the external world, including motor control and sensory processing.

In the present paper, we set the common coding theory, and the related theory of internal models, as a theoretical framework for understanding action-based effects on music perception. We conjecture that a focus on both inverse and forward modeling processes can provide a comprehensive view of how the human motor system and its actions influence music perception. In the domain of embodied music cognition, one typically refers to inverse modeling processes to explain action-based effects on music perception. Music spurs body movements that amount to expressive qualities, intentions, inner feelings, etc. Many of the musical elements that contribute to expressivity (e.g., dynamics, articulation, touch, phrasing, vibrato, rubato, etc.) directly relate to physical aspects of movement and space. Inverse modeling processes enable us to render (or decode) perceived patterns of musical expressivity into corresponding body movements. This corporeal mirroring process is responsible for listeners' tendency to ascribe intentions, inner feelings, etc. to music (Godøy, 2003; Leman, 2007; Cox, 2011). We want to extend this “traditional” embodied perspective to the role of the human body in music cognition with a focus on forward modeling processes. From this perspective, it is not about how the body resonates with the music, but rather about how predicted sensory outcomes of planned or performed actions can be projected onto the perceived music. Recently, there has been a proliferation of studies addressing the role of forward models in action-based effects on visual, auditory, and somatosensory perception. In the domain of visual perception, several papers review action-based effects on visual perception (Schütz-Bosbach and Prinz, 2007; Shin et al., 2010; Witt, 2011; Halász and Cunnington, 2012). Currently, such a review of studies investigating action-based effects on auditory perception does not exist. An important goal of the present paper is to provide such a review of studies in support of the proposed theories and principles.

The paper is structured as follows. In section 2, we argue that sensory-motor association learning can be considered a central mechanism underlying the development of internal models. Accordingly, we claim that the ability to predict the auditory consequences of one's actions, which is one of the core mechanisms of action-based effects on perception, depends on previous acquired sensory-motor associations. Further in that section, we define the concepts of temporal contiguity and probabilistic contingency as two main principles underlying associative learning processes. Additionally, we discuss musical instrument playing as a special but highly illustrative case of sensory-motor association learning. In section 3, we provide extensive empirical evidence for the claim that the principle of motor resonance, inherent in inverse models (section 3.1), together with auditory predictions generated by forward models (section 3.2), can modulate auditory perception. Also, we demonstrate that deficits in the motor system may have impaired auditory perception as a consequence (section 3.3). To conclude, an extensive discussion is presented in which we advocate a radical approach to embodied music cognition based on dynamical systems. Moreover, we pinpoint music as an ideal study object to extend this approach based on dynamical systems to embodied cognition, as it incorporates expressivity, introspection (affect, motivation, intentions, metacognition, etc.), and social interaction as crucial components.

## 2. Associative learning

Above, we outlined the common coding of action and perception as a core mechanism underlying people's engagement with music (motor control and sensory processing). However, this account does not address the question of how action and perception become integrated. We advocate that this integration is established, in large part, through associative learning processes. The study of these processes can be traced back to the philosophy of Aristotle who stated that things that occur near each other in time and/or space are readily associated (i.e., law of contiguity). During the Enlightenment, these ideas were further developed by the Associationist School (e.g., David Hume, John Locke, John Stuart Mill, etc.). In the nineteenth century William James stated, as an elementary law of association, that “when two elementary brain-processes have been active together or in immediate succession, one of them, on reoccurring, tends to propagate its excitement into the other” (James, 1890, p.566). In the late 1940s, this principle was paraphrased in Hebb's law “neurons that fire together wire together.” A more recent account is the theory of associative sequence learning (ASL) introduced by (Heyes and Ray, 2000). The ASL theory suggests that imitation is mediated by associative processes that establish links between sensory and motor representations. This theory has been applied to the human mirror neuron system (MNS) in an attempt to reconsider its origin and function. The classical view on the MNS—as originated in the work of Gallese et al. (1996); Rizzolatti et al. (2001); Kohler et al. (2002)—is that it is an innate system, only marginally influenced by sensory-motor experience, and inherently codes the meaning of actions (e.g., goals, intentions, etc.). This view was soon adopted to explain various important psychological and social functions, such as action understanding, learning by imitation, empathy, and social interaction. However, critical voices have been raised in opposition to this classical view, in particular to the idea that mirror neurons are adapted by evolution to directly and consistently encode action goals (Hickok, 2009; Heyes, 2010; Catmur, 2012). The alternative view—what Heyes (2010) termed the *associative hypothesis*—states that the development of the MNS is promoted by sensory-motor associative learning. Empirical evidence is provided in the context of music and dance. Haslinger et al. (2005) compared expert pianists with musically naive controls with fMRI while observing piano-playing and non-piano-playing finger movements. The results showed that the expert pianists exhibited stronger activation in brain areas associated with the MNS (inferior fronto-parieto-temporal region) compared to the control participants. Similarly, in the context of dance, Calvo-Merino et al. (2005) showed that activation in brain areas related with the MNS in expert dancers (classical ballet and capoeira) was higher when they observed a familiar dance style. In conclusion, the associative hypothesis states that, through systematically repeated experiences, sensory events are associated with particular motor acts and excitatory links between both are created, resulting in the development of “internal models.” Accordingly, when a sensory representation is activated, the corresponding motor representation is automatically co-activated (inverse modeling), and vice versa: when an action is merely planned or executed, the corresponding sensory representation is automatically co-activated (forward modeling). As will be explained further in Section 3, both inverse and forward modeling processes can contribute to action-based effects on auditory perception.

An important challenge of future research is to further identify the neural substrates underlying associative learning processes. Studies pinpoint the cerebellum (Imamizu and Kawato, 2009; Timmann et al., 2010), the striatum—an input nucleus of the basal ganglia—(Pasupathy and Miller, 2005; Williams and Eskandar, 2006; Lalazar and Vaadia, 2008; Melcher et al., 2012), prefrontal areas (Deiber et al., 1997; Bangert and Altenmüller, 2003; Pasupathy and Miller, 2005), the supplementary motor area (Pasupathy and Miller, 2005), and the premotor cortex (Deiber et al., 1997; Schubotz, 2007; Chen et al., 2009; Imamizu and Kawato, 2009) as important neural structures underlying association learning leading to the development of internal models and predictive mechanisms. In the field of music research, evidence suggests that the striatum is involved in prediction and anticipation. Grahn and Rowe (2013) assessed the role of the putamen—one of the two nuclei that make up the striatum—in beat prediction. Their findings show that the putamen becomes active only after having established a predictable sense of the beat. Accordingly, they conclude that putamen activity reflects the process of internally generating a model of the stimulus rhythm. In a study of Leaver et al. (2009), anticipatory/predictive imagery of musical melodies was shown to be associated with activation in a variety of cortical (frontal and parietal) and subcortical (basal ganglia and cerebellum) structures. Interestingly, different neural substrates underlay different stages of development of learned conditional associations between melodies (“moderately learned” vs. “well-learned”). Findings show that the supplementary motor area and the basal ganglia (putamen) are particularly important in early and moderate stages of learning, while the frontal cortex seems to dominate end stages (cf. Pasupathy and Miller, 2005). These dynamics in neural activation involved in sensorimotor association learning characterizes motor skill learning in general. Studies have demonstrated that the recruitment of distributed brain regions in the process of acquiring motor skills depends on the type of motor task (motor sequence learning vs. motor adaptation) and on the stage of learning (fast learning, slow learning, consolidation, automatization, retention) (Ungerleider et al., 2002; Luft and Buitrago, 2005; Doyon et al., 2009).

### 2.1. Continuity and contingency

Auditory-motor association learning—i.e., the acquisition of knowledge of sound-movement relationships—is modulated by both temporal “contiguity” and probabilistic “contingency” (Cooper et al., 2012). “Contiguity” refers to the proximity of two events (e.g., movement and sound) in time and space. The concept originates in Aristotle's law of contiguity, stating that things that occur near each other in time and/or space are readily associated. It is not, however, the case that association learning occurs every time two events are linked together in time or space. Instead, it is necessary that the relationship between the events is predictable. “Contingency” refers to this degree of probability or the likelihood that two or more events belong together. In statistical terms, contingency is related to covariance, being a measure of how much two random variables change together.

Elsner and Hommel (2004) present two experiments in which the role of contiguity and contingency were investigated in the development of sensory-motor associations. Each experiment consisted of a training phase followed by a test phase. In the training phase, participants learned action-effect associations by repeatedly pressing keys (action) triggering corresponding tones (effect). In the subsequent test phase, tones were presented and participants were asked to make speeded responses to these stimuli by pressing keys either in a consistent fashion (i.e., action-effect mapping as in the training phase) or inconsistent fashion (i.e., other action-effect mapping as in the training phase). If an action-effect association was established in the training phase, then participants were expected to respond faster in an acquisition-consistent fashion than in an acquisition-inconsistent fashion. In the training phase of Experiment 1, the contiguity between action and effect was systematically manipulated by adding an increasing delay between the two (50, 1000, and 2000 ms). In the test phase, participants responded faster in acquisition-consistent test blocks compared to acquisition-inconsistent test blocks when action-effects training delays were 50 or 1000 ms. Accordingly, association learning seemed to be successful only with action-effect delays of up to 1000 ms, signaling an effect of contiguity in association learning. In the training phase of Experiment 2, the contingency between action and effect was systematically manipulated by varying the relative frequencies of the presence or absence of tones with corresponding keypresses. Again, it was shown that the acquisition-consistency effect in the test phase was affected by the contingency of action and effect in the training phase. Together, these findings show that both the contiguity and contingency between actions (here, keypresses) and auditory events (here, sinusoidal tones, MIDI marimba/flute tones) are important in the process of acquiring sensory-motor associations.

An interesting experimental paradigm in which contiguity and contingency could be further investigated is the counter-mirror sensory-motor training paradigm (Cook et al., 2010). In this paradigm, previously established associations between motor and sensory events are manipulated by repeatedly pairing the observation of an action with the execution of another action. One typically finds (e.g., by measuring neural responses, or reaction times) that the original sensory-motor association gets weakened, depending on the principles of contingency and contiguity. This paradigm has been applied to visual-motor learning processes, but not yet to auditory-motor learning processes. However, the paradigm offers unique possibilities to study for instance how counter-mirror training can alter auditory-motor links established in musical instrument playing.

### 2.2. Musical instrument learning

Learning to play an instrument can be considered a special, highly illustrative case of sensory-motor association learning in which action and perception become intricately interwoven. The act of playing an instrument can be considered as a goal-directed, intentional act (Dalla Bella and Palmer, 2011). Ultimately, the goal of playing a musical instrument is to produce a certain sound. However, in order to reach that goal, one first needs to obtain knowledge about the relationship between the actions afforded by the musical instrument, and the auditory consequences of these actions. This knowledge is gradually acquired by exploring and manipulating the possibilities afforded by the instrument using (at first) arbitrary actions that lead to (at first) unexpected auditory events (Hommel, 2003). In that process of exploration and interaction, one systematically and repeatedly associates performed actions with heard sounds, and internal models are developed as a result, capturing the relationship between actions and sound. For example, in the case of the piano, one starts to understand that the key-to-pitch mapping is functionally organized (left-right motion corresponds to low-high pitch), or that depressing the sustain pedal creates a legato effect. At that point, playing a musical instrument may become a goal-directed act, in the sense that performers have the ability to intentionally produce certain sounds by performing certain actions. Additionally, it must be noted that the process of exploration in which action and perception mutually interact, is a continuous process throughout the life of a music performer. It incorporates aspects of creativity, intuition and surprise, and can in itself be a “raison d'être” of playing an instrument (cf. Sudnow, 1978).

A large body of empirical studies exist that support these ideas. For example, it has been shown that when people are trained to play a musical instrument, auditory-motor linkages are developed as a result of that training (Pascal-Leone, 2001; Bangert and Altenmüller, 2003; Lotze et al., 2003; Lahav et al., 2005; D'Ausilio et al., 2006; Lahav et al., 2007; Hyde et al., 2009; Herholz and Zatorre, 2012). Also studies have shown that during passive music listening, trained musicians exhibit stronger auditory-motor couplings compared to non-musicians (Haueisen and Knösche, 2001; Gaser and Schlaug, 2003; Baumann et al., 2007). This supports the idea that auditory-motor linkages are established by intensive training which involves long-term skill acquisition and the repetitive rehearsal of the same skills (Brown and Palmer, 2012, 2013).

It is evident that sensory-motor association processes are important for voluntary action control, as in musical instrument performance (Hommel, 1997, 2003; Elsner and Hommel, 2001). However, more important in the light of the present paper is the idea that sensory-motor relationships, and the integration of these relationships into internal models, may influence perceptual processes and accordingly shape the musical mind. In the following sections, we will discuss empirical evidence demonstrating that sensory-motor association learning, with musical instrument training as a special case, may lead to action-based effects on auditory perception.

## 3. Empirical evidence: a review

### 3.1. Inverse model: perception → action

Inverse models enable us to predict the motor commands that are required to achieve a desired sensory state. It is obvious that this is of utmost importance when playing a musical instrument. But inverse models hold an important role in music perception as well, as they allow to predict and simulate the physical aspects of motion and space implied in the music. There is ample evidence that merely listening to sounds or music automatically triggers motor responses, as a function of their previously established associations [motor resonance (Schütz-Bosbach and Prinz, 2007), perceiving action (Hurley, 2008), etc.]. This has been shown in neurophysiological studies (Haueisen and Knösche, 2001; Bangert and Altenmüller, 2003; Gaser and Schlaug, 2003; Lahav et al., 2005, 2007; D'Ausilio et al., 2006; Baumann et al., 2007; Chen et al., 2008). Additionally, results from behavioral studies show that motor responses to sounds are typically faster when the specific sounds and actions have been repeatedly and consistently paired on previous occasions (Elsner and Hommel, 2001; Rusconi et al., 2006; Lidji et al., 2007; Trimarchi and Luzzatti, 2011; Stewart et al., 2013a,b). These findings provide support for the idea that an action becomes automatically activated (or, primed) as a result of the mere perception of the auditory consequences normally associated with that action.^1^ Other studies have focused on overt body movements that people make in response to music for music presented in visual form, or via motion imagery (Eitan and Granot, 2006; Leman et al., 2009; Caramiaux et al., 2010; Godøy, 2010, Kozak et al., 2012; Bernardi et al., 2013; Küssner, 2013; Lotze, 2013). These studies show that people can consistently translate acoustic properties of sound and music into body movements, although Küssner (2013) reports that musicians are more consistent (i.e., less varying) in visualizing sound and music by means of drawings. More important in the scope of the present article is the idea that the power of music to induce body movements in listeners implies that merely listening to music becomes a kinaesthetic experience. Musical groove is a relevant example of a musical quality that induces body movements in listeners (Janata et al., 2012; Stupacher et al., 2013). The notion of music-induced body movement may be related to two ideas showing how inverse models, and the related concept of motor resonance (or, motor simulation), can shape people's engagement with music and by extension the “musical mind.”

First, the recruitment of the body into the process of music listening causes a connection to be made between the music and the expressive qualities inherent to the movements that the music induces. The human body acts thereby as a mediator between physical phenomena (sensory and motor processes) and subjective, mental states (Leman, 2007). An interesting model to capture the subtle qualities of movement expressivity is the Effort/Shape model that originated in the Laban Movement Analysis (LMA) method (Laban, 1947; Laban and Ullmann, 1966). This model is particularly appropriate, as it provides an integrated conceptual system connecting a set of physical movement properties with expressive qualities (e.g., weight, flow, space, time, etc.). The model has been used in research to show how music-induced body movements correlate with verbal descriptors used by people to describe their perception of the music (Maes et al., 2014).

Second, it is interesting to note that music-induced body movements may instigate a sense of *imagined participation* with the production of the sound. This idea of imagined participation is addressed in a broad range of musicological studies with different terminology, such as *imagined activity* (Maus, 1988), *kinaesthetic empathy* (Mead, 1999), *imaginary agency* (Levinson, 2006), *simulated control* (Leman, 2007), and *active perception* (Krueger, 2009). What these accounts have in common is their reference to a direct, sensory-motor engagement with music, to how music literally “moves” people, and to how people feel immersed in, and resonate with, the physical sound energy. In that sense, motor resonance may create the illusion of taking part in the actual skillful production of the music, which would be impossible in real life. Musical motion, however, is not limited to purely physical movements of the human body. Schubotz (2007) provides an answer to the question of how people can simulate or anticipate events that could not be readily reproduced by their own motor system (e.g., rhythm of ocean waves, the flight of a mosquito, or an unfolding sequence of abstract stimuli on a computer screen). Schubotz demonstrates and explains that even abstract events—including auditory events—recruit our motor system (in particular the premotor cortex and its parietal projection areas) in order to support simulation and prediction processes (see also Southgate, 2013). Accordingly, the micro and macro dynamics and subtleties inherent in the musical textures and structures, as for instance in the “Clocks and Clouds” (1973) of György Ligeti or in electronic music productions (e.g., Infected Mushroom, Aphex Twin, etc.), can evoke a fascinating continuum of spatial imagery and motion, with which the listener may float along. Accordingly, motor resonance may generate an experience of flow, being a state of heightened focus and immersion, typically accompanied with intense feelings of enjoyment and creativity (Csikszentmihalyi, 1988). This aspect of motor resonance is an essential component of musical aesthetic experiences and is fundamental for shaping the “musical mind.” Additionally, it may be a factor that explains the ability of music to alter people's experience of space and time (Schäafer et al., 2013), and to contribute to people's general well-being (Croom, 2011).

### 3.2. Forward model: action → perception

As explained, forward internal models represent an information flow from action to perception, in the sense that they allow the prediction of the likely sensory outcome of a planned or executed action [cf. “perceptual resonance” (Schütz-Bosbach and Prinz, 2007), “active perception” (Hurley, 2008), etc.]. Research has pinpointed the cerebellum as a crucial locus for internal forward models (Wolpert et al., 1998; Blakemore et al., 2001; Knolle et al., 2012a; Ebner, 2013), presumably in interaction with other brain structures [e.g., prefrontal areas (Lappe et al., 2013)]. In this context, it is important to note that different predictive mechanisms exist which are supported by different brain systems. O'Reilly et al. (2013) for example differentiate between statistical and dynamic predictive models. Statistical models capture the stochastic probability that two or more events are associated—for example an action event and a reward or sensory event—and are developed over a history of discrete events. Alternatively, in dynamic forward models, the relation between two events is deterministic and predictions are computed via explicit reference to pre-learned environmental dynamics.

Studies have shown that predictive models are important for motor control (Wolpert et al., 1995; Hommel, 1997), as well as for the processing of sensory information coming from the external environment (Halász and Cunnington, 2012). In the present study, we focus on the latter in the context of auditory perception. We will discuss how sensory predictions generated by forward models may influence the perception of sound and music. It will be shown that sensory predictions can either attenuate, facilitate, or disambiguate auditory perception (cf. Halász and Cunnington, 2012).

#### 3.2.1. Attenuation

Performing an action for which one can predict the sensory consequences attenuates the perception of the actual sensory outcome, as reflected in self-reports and neuronal responses. In the domain of auditory perception, this phenomenon was first studied in speech production (Houde et al., 2002; Heinks-Maldonado et al., 2006). Later on, studies appeared in which the phenomenon of motor-induced suppression (MIS) was studied with tones generated by keypresses. Despite the fact that the tones and the actions that produce them (i.e., action-to-pitch mapping) are highly simple, a parallel can be drawn with musical instrument playing, like playing the piano, trumpet, etc.

A study conducted by Aliu et al. (2009) demonstrates that the auditory response to tones generated by self-produced keypresses is attenuated relative to the response following passive listening to the same tones. However, because self- and externally-generated tones were presented in separate blocked conditions, it could not be ruled out that the observed attenuation effect was modulated by differences in contextual task demands (e.g., allocation of attention, arousal, etc.). To clarify this matter, Baess et al. (2011) mixed self- and externally-generated tones within blocks. The results of this study yielded an even larger attenuation effect for self-generated tones than that observed in the blocked condition. Also, Timm et al. (2013) conducted a study to further investigate the relationship between attention and the effects of motor prediction in perceiving auditory stimuli. The study adapted the mixed paradigm of Baess et al. (2011) and additionally incorporated different conditions in which attention was allocated to either the sound, the motor act, or to visual stimuli. Findings of this study demonstrated that an attenuation effect for self-generated sounds was independent from the allocation of attention. Other studies investigated whether the attenuation of auditory action effects occurs when actions are merely observed, instead of being self-generated. Sato (2008) hypothesized that, if there is a human mirror neuron system that codes a bidirectional association between action execution and action perception, then the mere observation of (well-learned) actions leading to a certain auditory event should bring about a similar auditory attenuation effect as when the action is self-generated. The results of this study confirmed this hypothesis, as similar auditory attenuation was observed for self-generated and merely observed sound-producing actions. However, this finding was later contradicted in a study by Weiss and Schütz-Bosbach (2012), using a comparable experimental protocol as in Sato (2008). They compared auditory action effects for self-generated actions, observed unanticipated actions, and observed anticipated actions. The results showed that the attenuation of a sound is significantly higher when the sound-producing action is self-generated compared to merely observed. Moreover, this effect was shown to be independent of whether the observed action could be anticipated or not. This finding raises questions about the role that forward internal models play in the prediction mechanisms underlying action effects on auditory attenuation (cf. Sato, 2008). More research is needed to clarify this point. In a last study we address here, Knolle et al. (2012b) examined whether auditory attenuation is a function of the degree of predictability of the self-generated sound. The result of this study indicated a lowering of the attenuation effect when self-generated sounds deviate from the expected outcome.

Together, these and similar studies (Baess et al., 2008; Hughes et al., 2013a,b; Jones et al., 2013; Loehr, 2013; Sanmiguel et al., 2013) provide strong evidence in support of the existence of an internal, motor-based prediction mechanism that can modulate auditory perception. Planning or executing an action causes a copy of the motor command to be made (i.e., “efferent copy,” or “corollary discharge”), which enables a prediction of the auditory outcome of that motor command. A comparison between the prediction and the actual auditory input (“reafference input”) leads to a small prediction error, and subsequently to a minimal response in the auditory cortex reflecting an attenuated perception (Aliu et al., 2009). This mechanism enables to discriminate between auditory inputs that are a consequence of our own actions and those that reach us from the external world. It is important to consider that this mechanism requires (learned) internal models about the relationship between sensory and motor representations. Only recently, studies have started to unveil the neural substrates of motor-based sensory prediction (Nelson et al., 2013; Roussel et al., 2013). However, more research is needed in order to obtain a full picture of the neural mechanisms underlying the action effect of auditory attenuation.

#### 3.2.2. Facilitation

Manning and Schutz (2013) examined to what extent “moving to the beat” objectively improves timing perception. They presented participants with sequences of 16 isochronous tones divided into groups of four followed by a probe tone. In the last group, the second, third, and fourth “tones” were silent (i.e., timekeeping segment). The probe tone was “on-time” (i.e., sounding after the same inter-onset interval), slightly early, or slightly late. The task of the participant was to judge whether the final probe tone sounded “on-time.” In one condition, participants were asked to tap along with the beat, while they remained still in the other condition. The results show that late offsets were better detected when participants could move during the timekeeping segment. Additionally, it was found that “better” tappers (i.e., less variability) performed better on the detection task overall. In general, these findings confirm that movement may improve time perception. Iordanescu et al. (2013) obtained similar results using a standard temporal-bisection paradigm. Participants were presented with sequences of three brief clicks with the location of the second click randomly varied. Participants had to judge whether the second click was temporally closer to the first or the third click. In the “active” condition, participants initiated each trial themselves by pressing the space bar, while trials were externally generated in the “passive” condition. Again, in line with the results of Manning and Schutz (2013), people in the active condition demonstrated a higher auditory sensitivity to temporal intervals. Moreover, it was shown that this effect was not attributable to the tactile sensation from a keypress. It is interesting to note that the finding that body movement can enhance time perception has been picked up by research in the domain of human-computer interaction (HCI) design. Maes et al. (2012, 2013) present a dance application and a music conducting application aiming to enhance users' understanding of temporal musical structures by teaching them how to articulate these temporal structures into corresponding body movements (dancing, conducting).

In another study, Brown and Palmer (2012) investigated how motor and auditory learning contribute to auditory memory for music. Pianists were asked to learn melodies on a Musical Instrument Digital Interface (MIDI) piano keyboard in each of four conditions (auditory only, motor only, strongly coupled auditory-motor [i.e., normal performance], or weakly coupled auditory-motor [i.e., performing along with auditory recordings (acoustically similar or varying) without hearing their own feedback]). After learning, participants heard melodies (half target, half foils) in a subsequent recognition test and were instructed to indicate which melodies they had encountered in the learning conditions. It was found that motor learning (combined with strongly coupled auditory learning) enhanced auditory recognition beyond auditory learning alone. Results were explained by the ability of sensory-motor associations formed during learning to provide additional retrieval cues and to shape auditory perception through mental simulation of action plans.

#### 3.2.3. Disambiguation

Music may have a certain degree of ambiguity in terms of perceptual and/or affective content. As discussed below, studies indicate that it is possible for a listener to disambiguate this content by planning or executing body movements during listening. Forward models provide an appropriate explanation for this disambiguation effect (Halász and Cunnington, 2012). The planning or execution of body movements enables one to automatically predict the sensory consequences of these actions. Consequently, these predicted sensory states can be projected onto the auditory or musical material, which may guide (i.e., disambiguate) the corresponding perception. Some additional remarks need to be made, however. First, planning body movements does not only generate predictions of sensory states, but equally of subjective mental states related to affect and expressivity (e.g., valence, arousal, etc.). In that sense, it is equally possible that subjective states are attributed to the music (Thompson et al., 2005; Juchniewicz, 2008; Sedlmeier et al., 2011; Maes and Leman, 2013). Second, auditory or musical material doesn't necessarily need to be ambiguous in order for body movements to guide our perception in a specific direction. Music presents the performer and listener with a flood of different auditory cues and accents. Body movements can help selectively direct attention to certain cues, and accordingly to impose a certain structure onto the music. According to Urista (2003); Pierce (2007), body movements can help to isolate and explore musical elements as melody, beat, and structural levels. Hence, cue selection (and, cue identification) facilitated by body movement may refine music listening in general, and shape our perception and understanding of the music. Third, studies show that merely observing body movements, instead of actually planning or executing them, may equally influence perceptual and aesthetic judgments of the produced music (Thompson et al., 2005; Schutz and Lipscomb, 2007; Juchniewicz, 2008). Fourth, it is possible that executed or observed body movements modulate auditory perception instantaneously, i.e., at the moment one listens to the music (Thompson et al., 2005; Schutz and Lipscomb, 2007; Juchniewicz, 2008; Repp and Knoblich, 2009; Sedlmeier et al., 2011). Additionally, it is also possible that when one repeatedly pairs body movements to music, the resulting action-based effects on music perception may endure for a longer period of time, in the sense that the specific way of perceiving music may retain when merely listening to the music without the need to intentionally plan or execute the corresponding body movements (Phillips-Silver and Trainor, 2005, 2007; Maes and Leman, 2013). So by sensory-motor associative learning processes, music may become integrated with actions and more importantly, with the sensory and affective states inherent to these actions. It is a form of “evaluative conditioning” leading to effects of disambiguation and cue selection (Juslin and Västfjäll, 2008; Maes and Leman, 2013). Moreover, depending on the nature of the learning process (e.g., duration, continuity, contingency, etc.), these effects can be retained for different amounts of time.

In the following section, we discuss several studies that illustrate these effects of disambiguation and cue selection. Phillips-Silver and Trainor (2005, 2007) addressed the interaction between body movement and the perception of musical rhythm. The procedures of the experiments conducted in these studies contained a training phase and a subsequent test phase. In the training phase, infants were passively bounced (Phillips-Silver and Trainor, 2005), or adults bounced actively by bending their knees (Phillips-Silver and Trainor, 2007) on every second (duple) vs. every third (triple) beat of an ambiguous musical rhythm pattern. In the subsequent test phase, infants' listening preferences were tested for two auditory versions of the rhythm pattern (duple and triple form) (Phillips-Silver and Trainor, 2005). In Phillips-Silver and Trainor (2007), the adults were asked to listen to two auditory rhythms (duple and triple rhythm) and to select the one they thought matched what they heard during the training phase. The results showed that the preferences and interpretations were oriented toward the auditory stimulus that matched the metrical form of their movement training.

In a study by Naveda and Leman (2009) it was shown that Samba music has a polymetric ambiguity, whereas Samba dance patterns typically have binary tendencies. Accordingly, the authors suggest that “perception of samba may be movement-based in the sense that through self-movement (of the dancer in response to music) musical patterns get rhythmically disambiguated.”

In a study by Sedlmeier et al. (2011), it was shown that real or imagined body movements during music listening may codetermine music preferences. The experimenters activated or inhibited specific muscles of the participants whose innervations have been shown to be associated with positive and negative emotions. This was realized by instructing the participants to perform three specific kinds of body movements or actions (activating/inhibiting “smiling muscles,” vertical/horizontal head movements, and flexion/extension of the arms). Activation of the positively associated muscle groups during listening to music led to higher preference ratings for that music than activation of the negatively associated ones. This suggests that body movements, both real and imagined, may play an important role in the development of music preferences.

Su and Pöppel (2012) tested the hypothesis that the use of body movement is not merely a reaction to hearing rhythmic input, but could actively assist the processing of temporal structures in auditory events. They suggest that a self-initiated movement frequency, which is not tuned-in at first, could be attracted to one of the underlying periodicities of the presented sequence. Doing so guides the listener to start “hearing” the pulse at that level, forming a positive audio-motor feedback loop. The authors show that in the absence of overt movement, by contrast, this tuning process must then rely on the internal motor entrainment and/or the ability to analyze the sequence. Unlike musicians, non-musicians seemed to lack an effective internal motor simulation that entrained to the pulse when it was not regularly present at the rhythmic surface, nor did they possess additional musical knowledge as a compensatory strategy.

A study by Iversen et al. (2009) investigated how the perception of a simple rhythmically ambiguous phrase (i.e., a repeating series of two tones followed by a rest) depends upon its intrinsic metrical interpretation. Participants were asked to mentally place the downbeat on either the first or the second tone of the rhythmical phrase. Using magnetoencephalography (MEG) it was shown that different metrical interpretations evoked different neural responses, specifically in the upper beta range (20–30 Hz). This led the authors—given the suggested role of beta in motor processing—to the hypothesis that the motor system influences metrical interpretation of sound, even in the absence of overt movement. In another study, Maes and Leman (2013) addressed the question of whether expressive body movements can condition children's perception of musical expressiveness. They trained children with a happy or a sad choreography in response to music that had an expressively ambiguous character. Afterwards, the children's perception of musical expressiveness in terms of valence and arousal was assessed. The results suggested that the expressive qualities of the movements they learned to associate with the music had a significant impact on how children perceived musical expressiveness.

In a study by Repp and Knoblich (2009), participants were asked to play pairs of octave-ambiguous (Shepard) tones which were a tritone apart. Although each tone of a pair is characterized by a specific pitch class (e.g., C - F#), they are ambiguous in pitch height. Participants were asked to play the pairs of tones by pressing corresponding piano keys or computer keyboard keys, either in left-to-right or right-to-left direction. Consecutively, they had to judge whether each pitch interval was rising or falling. Results showed that the participants gave significantly more “rising” responses when the order of keypresses was left-to-right than when it was right-to-left. Moreover, this effect was larger for pianists compared to non-pianist musicians, most likely because the specific pitch-to-sound mapping is stronger in pianists (Experiment 1). Additionally, the same effect was found when pianists merely observed another person pressing keys on a piano keyboard (Experiment 2).

Other studies have shown that merely observing musician's body movements can alter perceptual and aesthetic judgments of the produced music. Schutz and Lipscomb (2007) examined to what extent visual information of a marimba player's gestures can influence the perception of the duration of the produced tone. For the experiment, video recordings were made of a marimba player performing a series of tones using two stroke types (“long” gesture and “short” gesture). The tones that were produced by both stroke types were acoustically indistinguishable. The visual and auditory components were separated from each other and fully crossed in order to create realistic musical stimuli. Then, participants were asked to indicate perceived tone duration by means of a 101-point slider. In an audio-only condition, no significant differences occurred between the ratings. However, in the audio-visual condition, participants rated the tones produced with “long” gestures as significantly longer than the tones produced with “short” gestures. In another study Thompson et al. (2005) showed that facial expressivity and expressive hand gestures of music performers (i.e., vocal and guitar performance) can influence listeners' auditory perception of musical dissonance, melodic interval size, and affective valence. Similar findings are provided by Juchniewicz (2008), showing that the type of physical movement exhibited by a piano player while performing a musical excerpt (i.e., “no movement,” “head and facial movement,” and “full body movement”) alters listeners' judgments of the piano performance in terms of phrasing, dynamics, rubato and overall musical performance.

### 3.3. Motor disorders

The previously discussed action-based effects on auditory perception were rooted in learned auditory-motor associations. Apart from that, another category of action-based effects can be distinguished. Several studies have shown that motor dysfunction leads to considerable changes in individuals' perception and recognition of auditory and musical features. Pazzaglia et al. (2008) claimed a causative link between auditory recognition and action execution. Working with apraxia patients (limb apraxia, buccofacial apraxia, or both), they showed that deficits in performing gestures are causally linked to the patients' inability to recognize these gestures by their mere sounds. In the study, apraxia patients were asked to listen to a sound and then choose from among four pictures the one corresponding to the heard sound. Limb and buccofacial apraxia patients were impaired in recognizing sounds linked to respectively limb and buccofacial human actions. The authors advocated that lesions in frontal and parietal brain areas, which are actively associated with deficits in execution tasks, were responsible for the observed gesture-comprehension deficits. Also, studies indicate that the perception of musical features is impaired by motor dysfunctions. Beste et al. (2011) demonstrated effects of movement deterioration on rhythm processing in Huntington's disease patients. While listening to music, patients exhibited weaker activations overall in brain areas involved in the assessment of musical rhythms (cerebellar structures). Also, a study of Parkinson's disease patients by Grahn and Brett (2009) found that basal ganglia dysfunction results in an impairment of the processing of rhythms that have a beat. However, as the authors discuss, it cannot be excluded that pathological factors other than movement deterioration may contribute to impaired rhythm processing. For instance dopamine depletion, typical for Parkinson's disease, has been shown to affect emotional processing (Lotze et al., 2009), which may further modulate the processing of rhythms. In another study by Lucas et al. (2013), impaired temporal information processing in Parkinson's disease patients has been ascribed to a deficit in the process of sensorimotor integration. These and other studies (see e.g., Grahn, 2012 for a review) demonstrate that rhythm perception involves a close link between auditory and motor processes. The existence of such links has been exploited for motor rehabilitation purposes in the domain of Parkinson's disease, Huntington's disease, and stroke. In this context, musical activities involving movement (control) and rhythm (perception) have been shown to improve general motor performance in Parkinson's disease patients (Nombela et al., 2013a,b) and stroke patients (Altenmüller et al., 2009). It would be interesting to investigate further to what extent improvements in motor skill benefit performance on perceptual tasks.

## 4. Discussion

Traditionally, body movements—whether performed by a music performer or by a listener—were considered as the mere output of internal cognitive processes that involved a system of symbolic representations. Only recently, empirical evidence has begun to appear indicating that the human motor system and its actions may actually modulate people's experience, perception, and understanding of sound and music. The present article was intended to provide a theoretical framework in which action-based effects on auditory perception may be understood. Additionally, the article serves as a review in which we investigate how the theory applies to recent empirical findings. The presented theoretical framework is centered around the common coding theory (Prinz, 1990; Hommel et al., 2001). The basic assertion of this theory is that the planning or execution of an action recruits the same sensory-motor brain areas as the mere perception of the sensory consequences of that action. We have argued that associative learning, in which actions and sensory states are repeatedly experienced together, are of crucial importance in order for action and perception to become integrated, and to form so-called internal models. These internal models contain inverse and forward components. Inverse models allow incoming sensory information to activate the motor codes associated with the production of that sensory state (cf. direct-matching hypothesis Rizzolatti et al., 2001). In contrast, forward models allow the sensory outcomes to be predicted from planned actions (Waszak et al., 2012). The combination of inverse and forward models regulate goal-directed motor control (Wolpert et al., 1995; Hommel, 1997), as well as the processing of sensory information coming from the external environment (Halász and Cunnington, 2012). We explained that both inverse and forward models contribute to action-based effects on auditory perception. Inverse models allow that mere listening to music results in the activation of motor codes, which is often manifested in overt movement responses (cf. motor simulation, motor resonance, action mirroring, etc.). These body movements are experienced and understood as intentionally, expressively, and semantically meaningful, and cause the music to be experienced and understood accordingly. Forward models have an impact on music perception in a different way. They allow us to make predictions about the auditory outcomes of planned or executed actions, which guide and shape the perception of sound and music. Predictions may either attenuate, facilitate, or disambiguate the perception of sound and music. Together, these findings show that the human motor system and its actions have an impact on music perception and cognition. It is tempting to conclude based on this evidence that the “musical mind” is fundamentally embodied. However, according to Wilson and Golonka (2013), the assertion that (music) cognition is embodied has more radical and far-ranging implications. They claim that “embodiment is not simply another factor acting on otherwise disembodied cognitive processes.” This would retain the traditional Cartesian view that the brain is in control and, in the case of people's engagement in musical activities, literally “runs the show.” Instead, “radical embodiment” encompasses a perspective on the body, the mind, and the environment as substantial elements of a dynamical system (Chemero, 2009). In essence, the term “dynamical system” points to a system of elements which are coupled, mutually interactive, and evolve over time (Thelen and Smith, 1998). An important feature of dynamical systems is the ability to self-organize. Order and coherence appear out of the mutual interactions of the elements of the system without the use of explicit instructions, representations, or symbols. The dynamical system approach can be applied to motor control and development (Turvey, 1990; Kelso, 1995; Thelen and Smith, 1998; Warren, 2006), as well as to cognition (Port and Van Gelder, 1995; Van Gelder, 1998; Beer, 2000; Chemero, 2009; McClelland et al., 2010; Shapiro, 2013). Music seems especially relevant as many musical activities—e.g., music production, dance, music listening—provide an ecological setting in which the intrinsic dynamics of action and perception can be studied (Bader, 2013a,b). Moreover, it is interesting to note that people's engagement with music involves not only sensory and motor components but also other components, such as “introspection”—referring to internal states that include affect, motivation, intentions, metacognition, etc. (Barsalou, 2009)—and “social interaction.” Currently, research on internal models focuses almost exclusively on sensory and motor processes. However, to explain people's interaction with music, and by extension with the world in general, it is necessary to include aspects of introspection and social interaction into theories on internal models. The integration of these aspects into the present theoretical framework can deepen our understanding of music, and of the musical mind as fundamentally embodied. In the following paragraphs, we briefly discuss these two components.

### 4.1. Musical expressivity

An important aspect of people's engagement with music—whether in listening to music or the actual production of music—is musical expressivity. The musical elements that are said to constitute musical expressivity are manifold: dynamics, articulation, touch, phrasing, vibrato, etc. In the case of music production, musical expressivity is often—but not exclusively—related to the contents of the composition, and the main task of the musician is to render the composition into sound. Of course there is always a certain degree of interpretation and expressivity from the performer's side. Music performance however does not necessarily rely on a pre-composed score, as in the case of improvisation or jam sessions, where music may be created for the sake of exploring different sounds, rhythms, dynamics, etc. Apart from whether music is the result of playing a composition or improvisation, what is conspicuous about many of the various elements contributing to musical expressivity is that they directly relate to their physical origin, namely the body movements that produced the music (Repp, 1993; Shove and Repp, 1995; Johnson, 1997; Godøy, 2003; Leman, 2007; Cox, 2011). Accordingly, musical expressivity can be said to appeal to, at least to some extent, *kinaesthetic sensations* related to the effort and shape of body movements (Laban, 1947; Laban and Ullmann, 1966). Further, this kinaesthetic sensitivity may be associated with subjective phenomena like feeling, emotion, intentionality, etc. (Leman, 2007; Cochrane, 2010; Sievers et al., 2013). In that sense, the human body has been considered as a mediator between sensory and motor processes and mental states (Leman, 2007). A similar role has been attributed to the body in the context of music listening. A listener is assumed to be able to decode—i.e., identify, imagine, or even physically render—the elements of musical expressivity that relate to physical motion and space, based on their own action repertoire and notion of space. This kinaesthetic sensitivity may be related to subjective mental aspects of feeling, emotion, intentionality, etc. In the same way as planning or executing an action enables people to make predictions of the sensory consequences of that action, it is possible to make predictions of the consequences on a mental level (e.g., feeling, emotion, intentionality). Accordingly, it is reasonable to assume that the predictions of mental states modulate the perception of musical expressivity. It is only recently that empirical support for this idea emerged (Sedlmeier et al., 2011; Maes and Leman, 2013). Also, it has been shown that the visual observation of performers' body movements influences people's perception of musical expressivity (Davidson, 1993; Thompson et al., 2005; Juchniewicz, 2008). These findings provide support for including expressivity in theories of forward modeling applied to music perception and cognition. According to current theories of internal models, we have reason to believe that the relationship between mental states and action works in the opposite direction as well (cf. inverse models). In that sense, a subjective state coupled to music is assumed to modulate motor responses to music. Support for this idea is given in a study of Van Dyck et al. (2013). According to the current view, internal models guide goal-directed behavior as well as sensory processing. In that sense, internal models are the basic constituents of people's interaction with the outside world. We advocate that this view should be broadened by integrating other aspects of introspection (affect, motivation, intentions, metacognition, etc.). Musical behaviors provide opportunities to study interactions between sensory, motor and introspective processes, and the way these components become associated with each other. The current view of embodied music cognition considers introspection as a result of motor simulation processes (Leman, 2007). In other words, music induces body movements, which consequently trigger subjective aspects of feeling, emotion, intentionality, etc. We advocate that the relationship between body and mind may be bidirectional, as aspects of introspection may also influence motor responses to music.

### 4.2. Social interaction

In daily life, much of what we do and experience happens in a social context. A paramount example is people's engagement with music, as in music ensemble playing (Bastien and Hostager, 1988; Seddon, 2005), or when people dance together in a club or festival. These activities can be considered as forms of joint action involving coordinated actions, shared intention, shared attention, shared representations, etc. (Keller, 2008; Goebl and Palmer, 2009; Loehr and Palmer, 2011; Obhi and Sebanz, 2011; Pacherie, 2012; Phillips-Silver and Keller, 2012). Joint action in the context of music playing and dance has been shown to promote social behavior (Kirschner and Tomasello, 2010) and to establish a heightened sense of agency and a sense of we-ness (Pacherie, 2012). Also, studies show that the social context may modulate people's experience and perception of music (Egermann et al., 2011; Liljeström et al., 2012). Currently, a major line of research is devoted to the study of joint action in order to unveil the underlying mechanisms. Accumulating evidence suggests that these mechanisms are similar to the ones involved in individual voluntary motor control and information processing. Accordingly, internal models containing an inverse and forward component may explain how people manage to dynamically adapt to changes in each other's behavior. Inverse models are important for rendering desired joint action sensory outcomes into particular action plans. Supplementary forward models facilitate to anticipate (predict) the sensory consequences of one's own and other's actions.

Our discussion of the components of “introspection” and “social interaction” indicates that musical activities involve a high-dimensional dynamical system in which the body, the mind, and the external environment are continually and mutually interacting. In the case of musical instrument playing, music can be considered as the result of a dynamical interaction between the musicians' motor and sensory system, the constraints and opportunities of the pre-composed musical notation, the musical instruments and the social environment, and the musicians' intentions, personality, mental states, etc. The system in which these components interact is an open system, in the sense that no individual component has causal priority in generating the music (Thelen and Smith, 1998). It is possible, however, that the weight of the individual components on the produced sound varies depending on the specific musical activity (e.g., musical improvisation, historical informed music performance, jam session with an emphasis on social interaction, etc.). Similarly, music listening can be considered as a dynamical process, in which the experience, the perception, and the understanding of music is guided and shaped by the intrinsic dynamics of the body, the mind, and the external environment. In conclusion, adopting a fundamental embodied approach to music cognition requires us to consider music performance—involving motor coordination, control, and development—and music cognition as dynamical processes. The integration of theories on internal models and theories on dynamical systems can thereby enhance our understanding of how our body, mind, and the external environment interact in our engagement with the act of music.

### Conflict of interest statement

The authors declare that the research was conducted in the absence of any commercial or financial relationships that could be construed as a potential conflict of interest.

## Footnotes

^1^see Cox and Hasselman (2013) for some critical remarks on typical effect-priming studies.
